# Injectable homologous platelet-rich plasma, alone or in combination with oral omega-3 supplementation, for treating keratoconjunctivitis sicca in dogs

**DOI:** 10.14202/vetworld.2025.1262-1273

**Published:** 2025-05-21

**Authors:** William dos Santos Villa, João Victor Goulart Consoni Passareli, Giovana José Garcia Estanho, Marco Aurélio da Cruz Nobre Gomes, Gisele Alborghetti Nai, Cecília Laposy Santarém, Silvia Franco Andrade

**Affiliations:** 1 Postgraduate Program in Animal Science, UNOESTE, Presidente Prudente, SP, Brazil; 2 Department of Veterinary Ophthalmology, Veterinary Hospital, UNOESTE, Presidente Prudente, SP, Brazil

**Keywords:** canine ophthalmology, dry eye disease, keratoconjunctivitis sicca, ocular inflammation, omega-3 fatty acids, platelet-rich plasma

## Abstract

**Background and Aim:**

Keratoconjunctivitis sicca (KCS) is a chronic inflammatory condition of the canine ocular surface primarily caused by immune-mediated destruction of lacrimal tissues. Platelet-rich plasma (PRP) is known for its regenerative and anti-inflammatory properties, while omega-3 (ω-3) fatty acids possess immunomodulatory effects. This study aimed to evaluate the efficacy of injectable homologous PRP (HPRP), alone or in combination with oral ω-3 supplementation, in improving clinical and histopathological parameters in dogs with KCS.

**Materials and Methods:**

Twenty-two dogs (44 eyes) with bilateral KCS were randomized into two treatment groups: HPRP (n = 22 eyes) and HPRP plus oral ω-3 (HPRPO; n = 22 eyes). Treatments were administered monthly for up to three sessions alongside topical lubricants. Ophthalmological evaluations – including Schirmer’s tear test-1 (STT-1), tear film breakup time (TBUT), fluorescein staining, cytology of the third eyelid gland, and conjunctival histopathology – were performed at baseline and at monthly intervals up to 6 months.

**Results:**

Both groups demonstrated significant improvement in ocular clinical signs and tear secretion. STT-1 values significantly increased from baseline in both groups (p < 0.05), without significant intergroup differences. However, TBUT values were significantly higher in the HPRPO group from month 3 onwards (p < 0.05). Cytological and histological analyses revealed a significant reduction in lymphocyte and neutrophil counts and an increase in goblet cell numbers in both groups, with greater improvement in the HPRPO group (p < 0.05). Earlier resolution of corneal ulcers and reduced ocular inflammation were observed in the HPRPO group.

**Conclusion:**

Injectable HPRP, particularly when combined with oral ω-3 supplementation, is an effective therapeutic modality for managing KCS in dogs. The combination therapy enhanced tear film stability, reduced ocular inflammation, and promoted epithelial repair more effectively than HPRP alone. These findings support the synergistic effect of ω-3 fatty acids with PRP in ocular surface restoration.

## INTRODUCTION

Keratoconjunctivitis sicca (KCS), also known as dry eye syndrome, is a prevalent ocular disorder affecting both dogs and humans. It is predominantly immune-mediated, characterized by chronic inflammation of the lacrimal glands and conjunctiva, resulting in destabilization of the tear film and subsequent corneal surface alterations. The disease affects both the aqueous component (quantitative KCS) and the mucin and lipid components (qualitative KCS) of the tear film. Clinically, KCS manifests as mucopurulent discharge, conjunctivitis, keratitis, corneal opacity, neovascularization, pigmentation, and corneal ulceration [1–4]. Early diagnosis and timely therapeutic intervention are essential for minimizing ocular surface damage and preserving visual function. The Schirmer tear test (STT) and tear film break-up time (TBUT) are commonly employed diagnostic tools [5, 6].

Management typically involves the use of topical tear substitutes and immunomodulatory agents that stimulate tear production. Calcineurin inhibitors such as cyclosporine, tacrolimus, pimecrolimus, and sirolimus are widely used; these act by inhibiting the calcineurin, c-Jun N-terminal kinase, and p38 pathways, ultimately increasing the expression of transforming growth factor β1 (TGF-β1) [7–10].

Platelet-rich plasma (PRP) has gained considerable interest in ophthalmology due to its high concentrations of regenerative factors, including TGF-β, vascular endothelial growth factor (VEGF), and fibroblast growth factor (FGF), which promote corneal epithelial repair [11–14]. PRP also contains cytokines, integrins, and platelet-derived Vitamin A, contributing to tissue regeneration and immunomodulation. PRP is easily prepared, cost-effective, and versatile in application. It may be sourced autologously (same individual), homologously (same species), or heterologously (different species) [15–34]. Therapeutic-grade PRP requires platelet concentrations 4–7 times higher than baseline levels in whole blood [32]. PRP is further classif-ied based on cellular composition into pure PRP, leukocyte-rich PRP, red blood cell-rich PRP, and formulations rich in both red blood cells and leukocytes [33]. It can be formulated into various pharmaceutical preparations, including eye drops, injectable solutions, and clot forms for ocular use [35]. Injectable PRP has shown efficacy in treating dry eye in both humans [16, 17] and dogs [11, 34], with documented improvements in tear production, TBUT, and clinical signs. Moreover, PRP has been effective in promoting corneal ulcer healing in animal models, including rabbits [18]. In dogs, injectable PRP has been suggested as a viable alternative to topical tacrolimus for the treatment of KCS [11].

Essential fatty acids, particularly omega-3 (ω-3) and omega-6 (ω-6), are known to exert anti-inflammat-ory effects and have demonstrated therapeutic benefit in managing dry eye symptoms in both humans and animals [36–39]. Oral supplementation with ω-3 and ω-6 fatty acids has emerged as a viable adjunct treatment for various tear-deficiency syndromes, including Sjögren’s syndrome and KCS [40, 41]. The principal ω-3 fatty acids include alpha-linolenic acid (ALA), eicosapentaenoic acid (EPA), and docosahexaenoic acid (DHA), with EPA and DHA exhibiting therapeutic effects across species. ALA is enzymatically converted to EPA through Δ-6-desaturase and elongase, and subsequently to DHA through elongase and Δ-4-desaturase activity [42]. However, this conversion pathway is inefficient (<10%) in both dogs and humans, necessitating direct dietary intake of EPA and DHA to achieve therapeutic levels [42, 43]. ω-3 fatty acids mitigate inflammation by reducing levels of tumor necrosis factor-α, interleukin-1β, interleukin-6, nuclear factor kB, and reactive oxygen species. In addition, they facilitate tissue repair by limiting inflammation and promoting collagen deposition, which can prevent excessive scarring [44–48].

Despite the growing interest in regenerative therapies for canine KCS, current treatment paradigms remain heavily reliant on immunosuppressive eye drops, which are often limited by variable efficacy, the need for long-term administration, and potential side effects. PRP has emerged as a promising alternative due to its regenerative and immunomodulatory properties, with preliminary studies demonstrating its potential to improve tear production and corneal healing. However, the majority of existing research has focused on topical PRP or autologous preparations, with limited investigations into the use of homologous injectable PRP. Furthermore, while ω-3 fatty acids have been shown to reduce ocular surface inflammation and improve tear film stability in both human and veterinary medicine, no published studies have assessed the synergistic effect of ω-3 supplementation in conjunction with injectable PRP therapy for KCS. The lack of comparative clinical data evaluating combined therapeutic strategies presents a significant gap in optimizing non-pharmacological approaches to managing this condition.

This study aimed to evaluate and compare the therapeutic efficacy of injectable homologous PRP (HPRP), administered alone or in combination with oral ω-3 fatty acid supplementation, in the management of canine KCS. Specifically, the study assessed changes in tear production, tear film stability, corneal ulcer resolution, ocular inflammation, and goblet cell density over a 6-month treatment period. By investigating both clinical and histopathological outcomes, this research seeks to determine whether the addition of ω-3 supplementation enhances the regenerative and anti-inflammatory effects of HPRP, thereby providing a more effective and integrative treatment protocol for KCS in dogs.

## MATERIALS AND METHODS

### Ethical approval and Informed consent

This study was conducted in accordance with ethical guidelines and was approved by the Ethics Committee on Animal Use of the Universidade do Oeste Paulista (UNOESTE) under protocol number 6433. In addition, it complied with the ARVO Statement for the Use of Animals in Ophthalmic and Vision Research. Written informed consent was obtained from the owners or legal guardians of all animals enrolled in this study.

### Study period and location

The experimental procedures were performed between March 2021 and March 2023 at the Veterinary Hospital of UNOESTE, located in Presidente Prudente, São Paulo, Brazil.

### Animals and experimental design

A total of 44 eyes from 22 client-owned dogs were enrolled, comprising 9 males (four neutered, five intact) and 13 females (six neutered, seven intact) of various breeds. The mean age and body weight of the dogs were 6.8 ± 3.7 years (range: 1–15 years) and 13.3 ± 11.0 kg (range: 3.1–43 kg), respectively. The dogs were randomly assigned to one of two treatment groups: (1) injectable HPRP and (2) injectable HPRP combined with oral ω-3 fatty acid supplementation (HPRPO).

Inclusion criteria consisted of a clinical diagnosis of KCS based on slit-lamp examination (Kowa SL-15, Japan), compatible ophthalmic signs (e.g., pigmentation, neovascularization, ocular discharge, corneal ulceration, and conjunctival hyperemia), and STT-1 values <15 mm/min and/or TBUT <20 s. Dogs diagnosed with neoplasms or receiving systemic/topical immunosuppressive agents were excluded from the study. All ophthalmic evaluations were performed by a single examiner (JVGCP).

### Treatment groups

#### HPRP group

**Figure 1:**
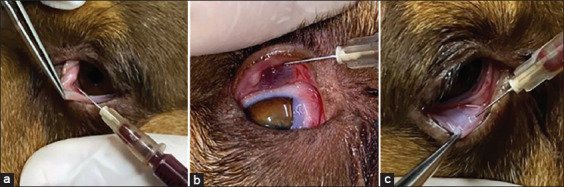
Homologous platelet-rich plasma application points. (a) Gland of the third eyelid. (b) Superior and (c) inferior palpebral conjunctiva.

This group included 11 dogs (22 eyes), consisting of 4 males (two neutered, two intact) and 7 females (three neutered, four intact), with a mean age of 9.7 ± 4.0 years (range: 5.0–15.0 years) and a mean body weight of 7.1 ± 2.4 kg (range: 3.6–12.1 kg). The breeds included six Lhasa Apsos, two Shih Tzus, two Yorkshires, and one Cocker Spaniel. Following instillation of proxymetacaine anesthetic eye drops (Anestalcon, Alcon), 0.3 mL of HPRP was administered per eye (0.1 mL each into the third eyelid gland, inferior conjunctiva, and superior conjunctiva) using an insulin syringe (Figure 1). Up to 3 monthly applications were performed, based on clinical improvement and STT-1/TBUT values. A topical ocular lubricant (sodium hyaluronate and carmellose sodium, Vetfresh Plus, Pet Society) was also applied twice daily for 6 months. Dogs showing no response after three HPRP applications were excluded and transitioned to standard immunosuppressive therapy.

#### HPRPO group

This group included 11 dogs (22 eyes): 5 males (2 neutered, 3 intact) and 6 females (3 neutered, 3 intact), with a mean age of 6.3 ± 2.8 years (range: 3.0–12.0 years) and a mean weight of 12.5 ± 7.8 kg (range: 3.1–30 kg). Breeds included Boxer, Fox Paulistinha, Labrador, Poodle, two Pugs, two Shih Tzus, and three mixed breeds. The HPRP injection protocol and lubricant regimen were identical to those used in the HPRP group (Figure 1). In addition, dogs received oral ω-3 capsules (Ograx, Avert): 500 mg/10 kg/day for dogs ≤10 kg or 1000 mg/day for those >10 kg, administered over 6 months.

In cases of bacterial ocular infection, tobramycin eye drops (Tobramicina 3 mg/mL, Neo Química, Brazil) were prescribed 4 times daily. For inflammation, sodium diclofenac eye drops (Still, Allergan) were administered 3 times daily for 15 days.

### HPRP processing

HPRP was prepared at the clinical analysis laboratory of the UNOESTE Veterinary Hospital, following the protocol described by Estanho *et al*. [11]. A single, clinically healthy, mixed-breed donor dog (7 years old, 34 kg) was used for all HPRP preparations. Whole blood (20 mL) was collected through sterile jugular venipuncture into tubes containing 3.2% sodium citrate. The first centrifugation was performed at 200 × *g* for 10 min, and 30% of the supernatant was discarded. The remaining plasma was transferred to a sterile Falcon tube and centrifuged at 400 × *g* for 10 min. The upper two-thirds, containing fewer platelets, were discarded as platelet-poor plasma, while the lower third, rich in platelets, was retained as PRP. From this, approximately 0.6 mL of PRP was extracted, with 0.1 mL applied to each ocular site as described.

### Ophthalmic evaluations

#### Clinical scoring and slit-lamp examination

Ophthalmic signs were evaluated monthly from baseline (M0) to 6 months (M6) using a portable slit lamp (SL-15, Kowa, Japan). Parameters scored included corneal pigmentation and neovascularization, ocular discharge, conjunctival hyperemia, and corneal ulcers. A severity scale was applied: 0 (none), 1 (mild), 2 (moderate), and 3 (severe).

#### STT-1

Performed without anesthesia, 0.5 cm of STT paper (Ophthalmos, SP, Brazil) was placed in the medial conjunctival sac for 1 min, and the wetted length was measured immediately. Values <15 mm/min were considered diagnostic of KCS [5].

#### TBUT

After instilling 1% fluorescein dye (Fludiag, Oftalmopharma, Brazil), dogs were manually blinked twice, and then the tear film rupture was observed under cobalt blue light. The time to break up was recorded using a stopwatch; the test was repeated twice and averaged. TBUT value ≤20 s was indicative of KCS [5].

#### Fluorescein test (FT)

Following fluorescein instillation and saline rinsing, the cornea was examined for staining to detect epithelial defects. Corneal ulcers were scored as 0 (absent) or 1 (present), and results were expressed as percentages.

### Cytological analysis

Cytological sampling of the third eyelid gland was conducted at M0, M3, and M6 using fine-needle aspiration. After topical anesthesia (proxymetacaine 5 mg/mL), the gland was exposed with forceps. Adrenaline and diclofenac drops were administered post-collection. Smears were methanol-fixed and stained using the May-Grunwald–Giemsa method. Lymphocytes, neutrophils, and squamous cells were quantified across five high-power fields (400×) using a Nikon Eclipse E200 microscope (Tokyo, Japan).

### Histopathological analysis

For the histopathological analysis of the conjunc-tiva, performed at M0 and M6, one drop of anesthetic eye drops containing 5 mg/mL of proxymetacaine hydrochloride (Anestalcon, Laboratory Alcon, São Paulo, Brazil) was instilled in each eye. Conjunctival biopsies were obtained using conjunctival scissors (HR, São Paulo, Brazil), with incisions made approximately 1.0 mm from the medial lower palpebral conjunctiva. The excised tissue fragments were positioned on paper strips (1 cm × 1 cm) [18]. Subsequently, adrenaline was administered, and sodium diclofenac eye drops (Still, Laboratory Allergan, São Paulo, Brazil) were instilled into the conjunctiva to minimize inflammation.

The collected specimens were fixed in formalin and subjected to two staining protocols: hematoxylin and eosin (HE; Dolles, São Paulo, Brazil) and periodic acid-Schiff (PAS; Merck, USA). The HE staining technique was employed to evaluate the numbers of polymorphonuclear and mononuclear neutrophils. The PAS technique was used to identify and quantify goblet cells. Cell counting on each histological slide was performed in five high-magnification fields using a 400× objective microscope (Nikon Eclipse E200). Light microscopy examination and image recording were carried out using a Leica ICC50HD microscope system (Wetzlar, Germany).

### Statistical analysis

Statistical analyses were performed using R software (R Development Core Team, 2022, Vienna, Austria). A two-way analysis of variance with Tukey’s *post hoc* test assessed temporal and intergroup differences for quantitative variables (e.g., STT-1, TBUT, and cell counts). The Kruskal–Wallis test and Dunn’s *post hoc* test were used for ordinal variables (clinical signs), while Chi-square tests were applied to categorical data (e.g., presence of ulcers). Data were presented as mean ± standard deviation (SD) for continuous variables and as medians (interquartile ranges) or percentages for ordinal/categorical variables. A significance threshold of p < 0.05 was applied. Effect sizes and confidence intervals were also calculated where appropriate.

## RESULTS

The initial mean platelet count was 249,154/mm^3^ (range: 160,000–350,000/mm^3^); after processing, the final mean platelet count increased to 1,124,192/mm^3^ (range: 459,000–1,771,000/mm^3^). Regarding the number of PRP applications required to achieve clinical improvement in KCS, in the HPRP group (n = 11), 4 dogs (36%) required three applications, 6 dogs (55%) required two applications, and 1 dog (9%) improved after a single application. In the HPRPO group (n = 11), 2 dogs (18%) required three applications, 6 dogs (55%) required two applications, and 3 dogs (27%) required only one application. Adverse reactions to PRP were observed in two dogs (one from each group, 4.5%) in the form of eyelid edema following treatment. These reactions resolved within a few minutes with the administration of diclofenac-based eye drops and application of a cold compress.

The median scores for the evaluation of ocular signs are presented in Table 1. Both treatment groups showed clinical improvement, including a reduction in corneal pigmentation, with statistically significant remission observed from month 3 (M3) to month 6 (M6) (p < 0.05). In the HPRP group, corneal neovascularization showed statistically significant remission from M5 to M6 (p < 0.05), whereas in the HPRPO group, this improvement occurred earlier, from M3 to M6 (p < 0.05). Secretion scores in the HPRP group showed statistically significant reduction from M4 to M6 (p < 0.05), while the HPRPO group demonstrated statistically significant improvement from M2 to M6 (p < 0.05). Conjunctival hyperemia resolved significantly from M3 to M5 in the HPRP group (p < 0.05), whereas the HPRPO group showed complete remission earlier, from M2 to M6 (p < 0.05).

**Table 1:** Median scores of clinical signs of the homologous platelet-rich plasma (HPRP) and HPRP with omega-3 fatty acid supplementation (HPRPO) treatment groups at the initial moment of diagnosis of KCS (M0), every 30 days, from moment 1 (M1) to final moment of treatment and assessment of KCS (M6).

Clinical signs	M0	M1	M2	M3	M4	M5	M6
Corneal pigmentation							
HPRP	1^a^	1^a^	1^a^	0^a^*	0^a^*	0^a^*	0^a^*
HPRPO	2^b^	1^a^	1^a^	0^a^*	0^a^*	0^a^*	0^a^*
Corneal neovascularization							
HPRP	1^a^	1^a^	1^a^	1^a^	1^a^	0^a^*	0^a^*
HPRPO	2^b^	1^a^	1^a^	0^b^*	0^b^*	0^a^*	0^a^*
Secretion							
HPRP	1^a^	1^a^	1^a^	1^a^	0^a^*	0^a^*	0^a^*
HPRPO	1^a^	1^a^	0^b^*	0^b^*	0^a^*	0^a^*	0^a^*
Conjunctival hyperemia							
HPRP	2^a^	1^a^	1^a^	0^a^*	0^a^*	0^a^*	0^a^*
HPRPO	2^a^	1^a^	0^b^*	0^a^*	0^a^*	0^a^*	0^a^*

Scores: (0) no change, (1) mild, (2) moderate, and (3) severe.

* p < 0.05 (Dunn’s test to compare moments with M0), ^a,b^p < 0.05 (Kruskal–Wallis test for comparing groups at each time point), KCS=Keratoconjunctivitis sicca

**Figure 2:**
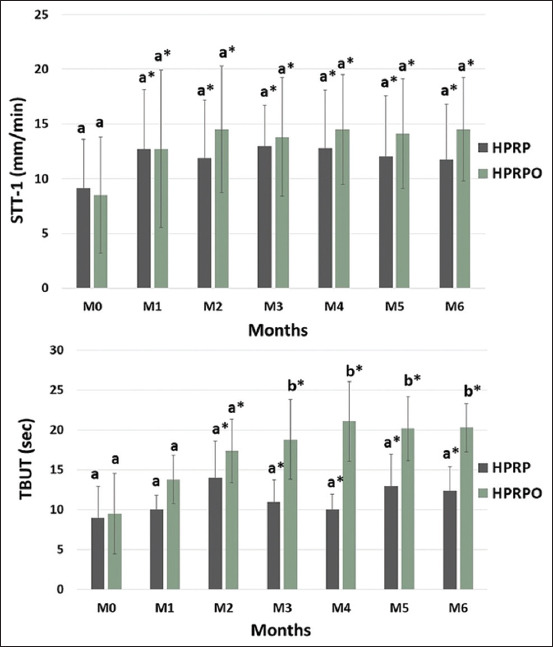
Mean and standard deviation of eye tests of the homologous platelet-rich plasma (HPRP) and HPRP+ oral omega-3 supplementation (HPRPO) groups at the initial moments (M0) and every 30 days, from moment 1 (M1) to moment 6 (M6). (a) Schirmer-1 tear test values in mm/min; (b) tear film break-up test values in seconds. *p < 0.05 (Tukey’s test to compare moments with M0), ^a,b^ p < 0.05 (Kruskal–Wallis test for comparing groups at each time point).

The mean and SD values for STT-1 and TBUT in the HPRP and HPRPO groups are presented in Figure 2. Both STT-1 (Figure 2a) and TBUT (Figure 2b) values significantly increased in both groups from M0 to M6 (p < 0.05). There was no statistically significant difference in STT-1 values between the two groups (p > 0.05). However, TBUT values from M3 to M6 were significantly higher in the HPRPO group compared to the HPRP group (p < 0.05).

**Figure 3:**
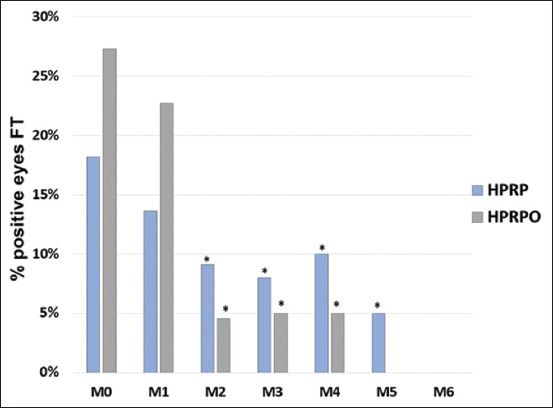
Percentage of eyes stained by fluorescein test in dogs with keratoconjunctivitis sicca (n = 22) in the homologous platelet-rich plasma (HPRP) and HPRP+ oral omega-3 supplementation groups. *p < 0.05 (Chi-square test).

**Figure 4:**
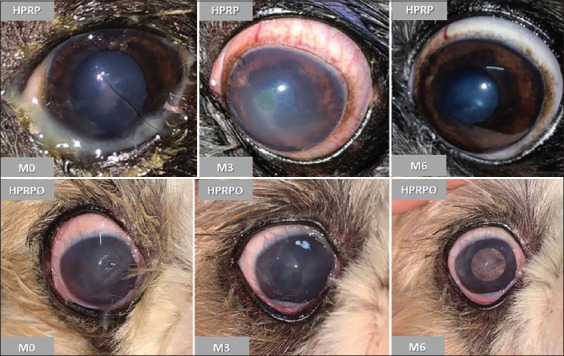
Homologous platelet-rich plasma (HPRP) group (dog no. 1) (Two applications of HPRP) and HPRP+ oral omega-3 supplementation group (dog no. 10) (Two applications of HPRP + 6 months of oral omega-3 supplementation) at the initial (M0), after 3 months (M3), and at the end of 6 months (M6).

The results of the FT are illustrated in Figure 3. A statistically significant reduction in corneal ulceration was observed from M2 to M6 in both groups (p < 0.05). In the HPRP group, fluorescein staining was absent from M6 onward, whereas in the HPRPO group, this resolution occurred earlier, with no staining observed from M5. The clinical signs in both groups are illustrated in Figure 4. At the end of the study period (M6), both groups demonstrated clinical improvement, including reductions in ocular secretion, conjunctival hyperemia, and complete corneal ulcer healing. Notably, the HPRPO group showed greater improvement in corneal pigmentation at 6 months.

**Figure 5:**
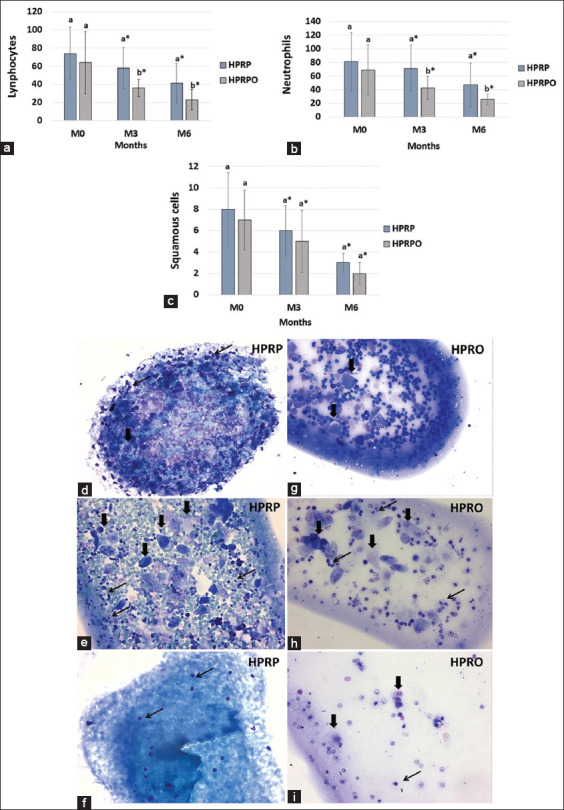
Cytology of the third eyelid gland performed at moments M0 (beginning), M3 (3 months), and M6 (6 months) after treatment with homologous platelet-rich plasma (HPRP) and HPRP+ oral omega-3 supplementation (HPRPO). Mean and standard deviation of counts of (a) lymphocytes, (b) neutrophils, and (c) squamous cells. Smears from HPRP group slides: (d) (M0) Increased number of lymphocytes, (e) M3: Moderate number of lymphocytes, and (f) M6: Small number of lymphocytes, neutrophils, and rare glandular cells. Smears from HPRPO group slides: (g) M0, Large number of lymphocytes; (h) M3, Moderate number of lymphocytes; and (i) M6, Small number of lymphocytes, neutrophils, and rare glandular cells. Thin arrows: inflammatory cells (lymphocytes and neutrophils). Thick arrows: glandular cells. Giemsa, 400× magnification. *p < 0.05 (Tukey’s test to compare moments with M0), ^a,b^p < 0.05 (Kruskal–Wallis test for comparing groups at each time point).

Lymphocyte, neutrophil, and squamous cell counts from cytological evaluation of the third eyelid gland are presented in Figure 5. A significant decrease (p < 0.05) in both lymphocyte and neutrophil counts was noted at M3 and M6 compared to M0 in both groups. A statistically significant difference between groups was observed at both M3 and M6, with the HPRPO group showing a more pronounced reduction in lymphocytes (Figure 5a) and neutrophils (Figure 5b). Compared to M0, squamous cell counts were significantly reduced at M3 and M6 (Figure 5c) (p < 0.05); however, the intergroup difference was not statistically significant at M6 (p > 0.05).

Smears from aspiration cytology slides in the HPRPO group showed a progressive reduction in lymphocyte and neutrophil counts from M0 to M6, indicating decreased inflammation in the third eyelid gland. At M0 (Figures 5d and 5e), a large number of lymphocytes were observed in the submucosa of the palpebral conjunctiva. At M3 (Figures 5f and 5g), moderate numbers of lymphocytes and occasional glandular cells were detected. By M6 (Figures 5h and 5i), only small numbers of lymphocytes, neutrophils, and rare glandular cells were present.

**Figure 6:**
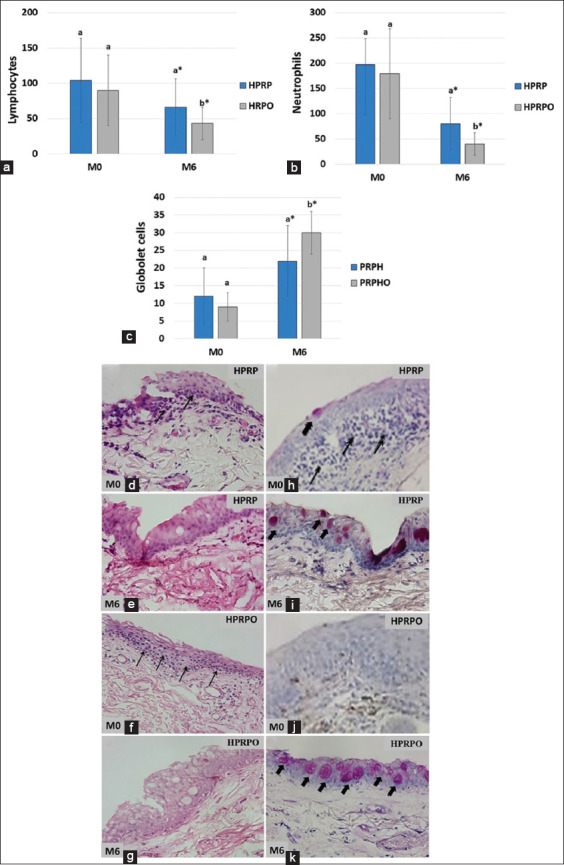
Histopathology of the palpebral conjunctiva of the homologous platelet-rich plasma (HPRP) and HPRP+ oral omega-3 supplementation (HPRPO) groups at M0 and M6. Mean and standard deviation of the counts of (a) lymphocytes, (b) neutrophils, and (c) goblet cells. Biopsy slides stained with HE (hematoxylin and eosin) 400× magnification showing: HPRP group (d) M0, inflammatory infiltrate in the submucosa (thin arrows); (e) M6, absence of inflammatory infiltrate; HPRPO group (f) M0, inflammatory infiltrate in the submucosa (thin arrows) and (g) M6, absence of inflammatory infiltrate. Biopsy slides stained with periodic acid-Schiff at 400× magnification showing: HPRP group (h) M0, absence of goblet cells, with presence of inflammatory infiltrate in the submucosa (thin arrows), and (i) M6, large number of goblet cells (wide arrows); and HPRPO group (j) M0, absence of goblet cells, with presence of inflammatory infiltrate in the submucosa (thin arrows) and (k) M6, large number of goblet cells (wide arrows). *p < 0.05 (Tukey’s test to compare moments with M0), ^a,b^p < 0.05 (Kruskal–Wallis test for comparing groups at each time point).

The results of the histopathological evaluation of lymphocyte, neutrophil, and goblet cell counts in the lower palpebral conjunctiva are presented in Figure 6. Compared to M0, both lymphocyte and neutrophil counts significantly decreased at M3 and M6 in both treatment groups. A statistically significant difference was noted between the HPRP and HPRPO groups at M6, with the HPRPO group showing a greater reduction in lymphocytes (Figure 6a) and neutrophils (Figure 6b). Goblet cell counts significantly increased at M6 compared to M0 (Figure 6c) in both groups. The increase in goblet cells was more pronounced in the HPRPO group and statistically significant (p < 0.05).

Biopsy slides stained with HE showed an inflammatory infiltrate in the submucosa of the HPRP group at M0 (Figure 6d), which was absent at M6 (Figure 6e). In the HPRPO group, inflammatory infiltrates were present at M0 (Figures 6f and 6g) but had resolved by M6. Biopsy slides stained with PAS in the HPRP group at M0 showed absence of goblet cells and presence of submucosal inflammatory infiltrates (Figure 6h), while at M6 (Figure 6i), a large number of goblet cells were observed. Similarly, in the HPRPO group, M0 slides (Figure 6j) revealed a lack of goblet cells and prominent inflammation in the submucosa, whereas M6 slides (Figure 6k) showed a marked increase in goblet cells.

## DISCUSSION

This study is the first to compare the therapeutic efficacy of injectable HPRP with and without oral ω-3 supplementation in dogs diagnosed with KCS, with the objective of enhancing regenerative cellular therapy. The final mean platelet concentration obtained during PRP extraction in this study was 1,124,192/mm^3^, which corresponds to a 4–7-fold increase relative to whole blood, consistent with established recommendations in the literature [11, 32].

Estanho *et al*. [11] reported that injectable HPRP typically requires two to three administrations for clinical efficacy, with only one dog (9%) responding to a single application. These results align with our findings. However, in the present study, the addition of oral ω-3 supplementation increased the percentage of dogs responding after a single PRP administration to 27% (3 dogs), thereby highlighting the potential benefit of combining ω-3 with HPRP therapy. The administration of PRP into the third eyelid gland and the upper and lower palpebral conjunctiva proved to be safe, with only two cases of mild hypersensitivity reactions – one in each treatment group – manifesting as transient eyelid edema. These mild reactions resolved rapidly following topical diclofenac administration and application of a cold compress, corroborating safety data reported in previous studies by Estanho *et al*. [11] and Latalski *et al*. [49]. Furthermore, no gastrointestinal side effects or adverse systemic reactions were noted with the 6-month administration of oral ω-3, consistent with previous findings by Lenox and Bauer [43].

Both treatment groups exhibited significant improvement in ocular clinical signs (Table 1) and healing of corneal ulcers; however, the HPRPO group demonstrated earlier clinical resolution. ω-3 fatty acids, particularly EPA and DHA, have known bioactive properties. EPA is a precursor to the E-series resolvins (RvE1 and RvE2), which possess anti-inflammatory and immunomodulatory effects, including downregulation of pro-inflammatory leukocytes and cytokine migration, leading to attenuation of the inflammatory response [47, 48]. In parallel, DHA is metabolized into D-series resolvins (D1, D2), protectins, and neuroprotectins – lipid mediators that modulate inflammation in both ocular and neural tissues [44–48]. The results of Silva *et al*. [38] further support our find -ings, demonstrating enhanced efficacy of topical tacrolimus in dogs with KCS when co-administered with EPA and DHA, thereby corroborating the benefits obser-ved with the HPRPO combination in the present study.

With regard to tear volume, as assessed by STT-1, our findings were comparable to those reported by Estanho *et al*. [11], and superior to outcomes reported with tacrolimus. Although both groups showed statistically significant increases in tear production from baseline, no significant differences were detected between the HPRP and HPRPO groups. Conversely, tear quality, as evaluated by TBUT, improved significantly in the HPRPO group from M3 to M6 relative to the HPRP group. This improvement is consistent with prior reports indicating that oral ω-3 supplementation can enhance tear stability by mitigating inflammation in the meibomian glands, increasing mucin secretion, and reducing tear evaporation [38–42].

The observed improvement in tear quality may be attributed to the overall enhancement of clinical signs and the accelerated resolution of corneal ulcers, thus potentiating the regenerative effects of PRP on the ocular surface [14, 15, 18, 19]. In addition, the significant increase in goblet cell density, which plays a vital role in mucin production, further supports the improvement in TBUT values observed in the HPRPO group [38–40].

Cytological and histopathological evaluations revealed a significant reduction in lymphocyte and neutrophil counts in both treatment groups, with the HPRPO group showing a more pronounced reduction. These findings are in agreement with previous studies by De Oliveira *et al*. [7] and Silva *et al*. [38] and suggest that EPA exerts a strong anti-inflammatory effect by being metabolized into RvE1, which activates macrophages and enhances phagocytic activity. This mechanism may synergize with the immunomodulatory actions of PRP to reduce inflammation and promote healing [7]. The concurrent decrease in inflammatory cell infiltrates and the increase in goblet cell numbers underscore the beneficial effects of combining ω-3 fatty acids with PRP in treating KCS.

Our findings further highlight the synergistic action of PRP, which contains high concentrations of growth factors such as TGF-β, VEGF, and FGF. These factors are known to accelerate corneal epithelial healing [14, 15, 18]. Moreover, PRP contains bioactive proteins, primarily derived from platelet alpha-granules, which exert potent immunomodulatory effects [15–19]. When administered in conjunction with ω-3 fatty acids – specifically EPA and DHA, which are naturally anti-inflammatory – the combination enhances tear film quality, supports collagen matrix deposition, stimulates fibroblast metabolism, and promotes keratinocyte proliferation. These biological effects collectively contribute to earlier improvement in ocular signs and enhanced corneal healing [38–45].

It is important to note that a high prevalence of brachycephalic breeds was observed in the study cohort. KCS is particularly common in brachycephalic dog breeds such as Pugs, Bulldogs, Shih Tzus, Lhasa Apsos, and Boston Terriers. These breeds are predisposed to excessive tear evaporation due to incomplete blinking and anatomical abnormalities in eyelid conformation and nasolacrimal duct drainage [50–52].

## CONCLUSION

This study demonstrated that injectable HPRP, particularly when combined with oral ω-3 supplementation, is an effective and safe therapeutic approach for the management of KCS in dogs. Both treatment groups exhibited significant improvement in clinical signs, tear production, and ocular surface health. However, the group receiving combined therapy (HPRPO) showed earlier clinical improvement, greater increases in tear film stability (TBUT), faster resolution of corneal ulcers, and a more pronounced reduction in ocular inflammation. Cytological and histopathological analyses confirmed a significant decrease in lymphocyte and neutrophil infiltration, along with an increase in goblet cell density, particularly in the HPRPO group, indicating enhanced mucin production and tear film quality.

The findings suggest that integrating oral ω-3 fatty acids with injectable HPRP therapy may potenti-ate the anti-inflammatory and regenerative effects of PRP, offering a viable adjunct or alternative to conventional immunosuppressive treatments for canine KCS. This approach may be especially valuable in dogs that are refractory to or intolerant of standard topical immunomodulators.

This is the first controlled study to compare injectable HPRP alone versus HPRP combined with oral ω-3 in canine KCS, employing a comprehensive clinical, cytological, and histopathological evaluation over 6 months. The use of HPRP, a safe, low-cost, and mini-mally invasive biological therapy, further underscores the translational relevance of this intervention.

The primary limitations include the modest sample size and breed variability, with a predominance of brachycephalic breeds, which may limit generalizability. In addition, long-term follow-up beyond 6 months was not conducted.

Future studies should evaluate the long-term durability of treatment effects, explore dose optimization of ω-3 supplementation, and assess the applicability of this combined therapy across larger and more diverse canine populations. Comparative studies with standard immunosuppressants such as cyclosporine and tacrolimus would also help define the relative efficacy and safety of HPRP-based regenerative protocols.

## AUTHORS’ CONTRIBUTIONS

WSV: Designed and conducted the study, performed all animal examinations and tests, analyzed the data, prepared graphs, figures, and tables, and drafted the manuscript. JVGCOP and GJGE: Performed all animal examinations and tests. GAN and MACNG: Performed cytological and histopathological analyses. CLS: Conceptualized the aim of the study, planned, supervised, designed, and performed the study, analyzed the data, and prepared the HPRP. SFA: Conceptualized the aim of the study, planned, supervised, designed, and conducted the study, performed all animal examinations and tests, analyzed the data, prepared graphs, figures, and tables, and drafted the manuscript. All the authors have read and approved the final version of the manuscript.
